# Barriers to efficient management of in‐home care: A qualitative content analysis

**DOI:** 10.1002/nop2.1161

**Published:** 2021-12-15

**Authors:** Parisa Sabetsarvestani, Fateme Mohammadi, Banafsheh Tehranineshat, Mostafa Bijani, Zhila Fereidouni

**Affiliations:** ^1^ Master of Nursing School of Nursing Fasa University of Medical Sciences Fasa Iran; ^2^ Chronic Diseases (Home Care) Research Center and Autism Spectrum Disorders Research Center Department of Nursing Hamadan University of Medical Sciences Hamadan Iran; ^3^ Community‐based Psychiatric Care Research Center Department of Nursing School of Nursing and Midwifery Shiraz University of Medical Sciences Shiraz Iran; ^4^ School of Nursing Fasa University of Medical Sciences Fasa Iran

**Keywords:** home care service, home health nursing, nursing care, qualitative research

## Abstract

**Aim:**

Inefficient management is one of the major barriers to development of in‐home care in the society. Accordingly, the present study aims to identify the barriers to efficient management of home care nursing using a qualitative approach.

**Design:**

The present study is a qualitative‐descriptive work of research.

**Method:**

Data were collected using semi‐structured, in‐depth, individual interviews with 19 nurses from November 2020 to May 2021. The collected data were analysed using Graneheim and Lundman's method.

**Results:**

The findings of the study were categorized into four main themes, namely lack of effective standards, ineffective interactions, inappropriate cultural/social context and professional issues, and 15 subthemes.

**Conclusion:**

In‐home care nurses in Iran experience various problems in their practice. Creating an appropriate cultural/social context in Iranian societies, providing the necessary infrastructure, including insurance, providing comprehensive, clear guidelines for in‐home care, encouraging teamwork and organizing workshops to promote effective interactions between the personnel and patients can improve the quality of in‐home care nursing.

## 1 INTRODUCTION

The ever‐increasing prevalence of long‐term diseases, disabilities and conditions caused by old age in recent decades has posed many countries with a variety of healthcare challenges (Sethi & Rani, 2017). Providing long‐term care to patients in clinical environments like hospitals and clinics not only imposes considerable costs on the healthcare system, but exposes patients and their families to various forms of emotional and psychological tension (Halevi Hochwald et al., 2020). As an innovative strategy, in‐home care has raised interest as a way to prevent or minimize the problems associated with prolonged hospitalization (Johannessen et al., 2020; Lindblad et al., 2018). In‐home care is an essential service which is provided in patients’ homes with the cooperation of patients and their families and has many benefits for patients, as well as the healthcare system (Wiig et al., 2018). In this way, Floridi et al. (2021) also state that socioeconomic condition is effect in the use of home care, so that people with unfavourable economic conditions cannot afford to use home care services and need more family and social support. Also, Brant et al. (2021) stated leaders should work with governments and ministries of health to promote home care and improve symptom management and alleviate suffering for patients.

### 1.1 Background

Among the most significant benefits of in‐home care are the following: providing timely, comprehensive care to patients, disease management, completing hospital services, reducing medical costs, decreasing the need for in‐hospital care, increasing the participation of patients and their families and raising patient satisfaction (Estabrooks et al., 2011; Kattouw & Wiig, 2019; Ree et al., 2019; Xiao et al.,^2017^). Accepted by many in today's world, in‐home care has paved the ground for expansion of healthcare services throughout the society (Estabrooks et al., 2015). Yet, studies show that there are certain obstacles to effective in‐home care, including caregivers’ poor competence, lack of a suitable infrastructure, cultural issues, insufficient inter‐professional coordination and cooperation, caregivers’ dissatisfaction, lack of acceptance in the society and inefficient management (Konetzka, 2020; Song et al., 2020; Wiig et al., 2018). One of the main barriers to development of in‐home care on a social level is inefficient management. It is clear that poor management and organization results in the dissatisfaction of caregivers, patients and patients’ families, as well as the society's reluctance to accept this type of care.

All of the above studies state although home care has numerous advantages, but there are still cultural, social and economic barriers to its implementation in many parts of the world. These barriers in Iranian society and culture due to religious and ethnic attitudes can be different from other places. Accordingly, there is a need for more research. In this way, qualitative research can play a significant role in exploring, describing and obtaining an in‐depth understanding of the management problems and challenges in the implementation of in‐home care in Iran. An understanding of the different aspects of in‐home care nurses and the current management issues in this area can help with a proper evaluation of the quality of this type of care. Considering the multi‐dimensional nature of the management challenges in in‐home care, qualitative content analysis is a proper method for investigating the subject. The present study was conducted to identify the barriers to efficient management of in‐home care.

## 2 METHODS

### 2.1 Design

The present study used a descriptive qualitative design which is an effective method to obtain insight into a research question and establish the subjects’ perception of who, what, place of events or experiences treatment (Doyle, et al., 2020). As the barriers to efficient in‐home care management in Iran are unknown, the researchers used the above‐mentioned approach. The reporting of the study was based on the consolidated criteria for reporting qualitative research (COREQ) checklist (Tong et al., 2007).

### 2.2 Sampling and recruitment processes

In this study, selected by purposeful sampling, 19 in‐home care nurses, who worked as caregivers at home, participated in the study. The study lasted from November 2020 to May 2021. The inclusion criteria were having at least 1 year's experience of in‐home care, being Iranian, speaking and understanding the Persian language, and the ability to provide appropriate and sufficient information on the subject. The authors try to select participation with maximum variation. Therefore, nurses were selected from a wide range of age, sex, marriage and educational level. In this study, 19 nurses worked in 3 home care services. Participants’ information is provided in Table 1.

**TABLE 1:** Descriptive characteristics of the participants

Participants	Sex	Marital status	Educational level	Work experience (years)
P1	Male	Single	Bachelor's degree in nursing	13
P2	Female	Married	Bachelor's degree in nursing	10
P3	Male	Married	Master's degree in nursing	18
P4	Female	Married	Bachelor's degree in nursing	2
P5	Male	Married	Associate's degree in Nursing	8
P6	Female	Single	Master's degree in nursing	7
P7	Male	Married	Master's degree in nursing	9
P8	Male	Married	Master's degree in nursing	10
P9	Female	Single	Master's degree in nursing	8
P10	Male	Married	Bachelor's degree in nursing	18
P11	Female	Married	Bachelor's degree in nursing	5
P12	Male	Single	Master's degree in nursing	15
P13	Male	Single	Bachelor's degree in nursing	3
P14	Male	Married	Bachelor's degree in nursing	2
P15	Male	Married	Bachelor's degree in nursing	2
P16	Female	Single	Bachelor's degree in nursing	13
P17	Male	Married	Master's degree in nursing	5
P18	Male	Married	Bachelor's degree in nursing	3
P19	Female	Single	Bachelor's degree in nursing	7

### 2.3 Data collection

Twenty‐three nurses were invited to participate in the study, but 4 nurses refused due to busy schedule, intensive shifts and spending time with their families. Therefore, data were collected through 19 semi‐structured individual interviews with 19 nurses. The participants were video called for interviews on WhatsApp at times which were convenient for them. The second author, who is an assistant professor of nursing and has led numerous qualitative studies, conducted and analysed all the interviews. Each interview started with a few general questions: “Can you describe your typical day of in‐home care?”, “Based on your experiences, what challenges and barriers adversely affect the quality of services provided during in‐home care?”, “What strategies do you suggest for improving the quality of in‐home care services?” and “How have the existing barriers affected your performance as a caregiver?” Next, based on the participants’ answers, follow‐up questions would be asked to increase the clarity of the information, for example “Can you explain further?”, “What do you mean by that?” and “Can you give an example?” The interviews were oriented around the main objective of the study. Each interview lasted from 38 to 52 min. Immediately after each interview, the first author would listen to the recorded interview several times and transcribe it. Immediate analysis of the data collected from each interview allowed the researchers to plan the next interview in view of the data they had obtained already from the previous interviews.

### 2.4 Data analysis

The collected data were analysed according to qualitative conventional content analysis. Thus, based on the explicit and implicit content of the meaning units, key points in the transcripts were extracted as open codes. The open codes were classified based on their similarities and differences and the process of abstraction continued until a theme emerged (Graneheim & Lundman, 2004). Accordingly immediately after each interview, the first author (PS) transcribed the recorded content. At the reading stage, the transcripts were carefully read line by line and the significant paragraphs were marked to stand out. The words, sentences or paragraphs that carried significance about the challenges to efficient management of home care were also selected as meaning units. Each meaning unit was assigned a code. At the next stage, the second author (FM) reviewed the transcripts and verified the unit meanings and codes. The codes that were found to be similar and homogenous were then merged to form categories. To confirm the reliability of the codes, the researchers reviewed the categories and compared them with the primary data. Eventually, in several joint meetings, after contemplating and comparing the categories, the research team extracted the obtained themes. MAXQDA software 10.0 R250412 was used to help data analysis and classification (Table 2, Example of data analysis process).

**TABLE 2:** An example of coding and development of subthemes and themes

Meaning units	Coding	Subthemes	Theme
“In in‐home care, what is missing is a clear established protocol which is based on scientific guidelines and evidence. Sometimes, I wonder if the procedures which I perform are scientific or not. There is a sense of indecision. I really don't know what instructions I should follow when I’m providing care” (P6).	Lack of protocol based on scientific guidelines	Lack of effective protocols for in‐home care	Lack of effective and comprehensive standards

### 2.5 Rigour

To test the trustworthiness of the data, the researchers applied Lincoln and Guba's criteria (Cypress, 2017). Accordingly, to ensure credibility, the researchers used prolonged engagement with data, member checking and peer debriefing. For member checking, 5 members of the nursing staff were asked to evaluate a copy of the encoded interviews. For peer‐checking, 7 experts were asked to examine the process of data analysis and verify the codes and categories. The dependability of the findings was enhanced by describing the methods of coding the concepts and themes and providing textual and audio data. Moreover, three members of the research team examined the findings separately and subsequently discussed them to resolve any disagreements towards ensuring dependability. To ensure the confirmability of the data, the researchers presented the participants with the encoded data and asked them to confirm the accuracy of the extracted categories and subcategories. By providing detailed information about the respondents’ characteristics, manner of conducting the interviews, and the methods of data collection and data analysis, along with documented examples of the participants’ quotes, the researchers increased the transferability of the study results. The study subjects were selected in view of the objectives of the study and in an unbiased manner. As soon as they were collected, the data were analysed to help the researchers be aware of the development of the research.

### 2.6 Ethical considerations

All participants gave written informed consent to participate in the study. The present study was conducted in accordance with the principles of the revised Declaration of Helsinki, a statement of ethical principles which directs physicians and other participants in medical research involving human subjects. Also, the study was approved by the local Ethics Committee of Fasa University of Medical Sciences, Fasa, Iran (IR.FUMS.REC.1399.141).

## 3 RESULTS

In this study, 19 nurses (12 male and 7 Female) participated with the clinical nursing experience of 2–18 years. The 4 main themes and 15 subthemes extracted from the data are shown in Table 3.

**TABLE 3:** Themes and subthemes extracted from content analysis

Themes	Subthemes
Lack of effective and comprehensive standards	▪ Lack of effective protocols for in‐home care ▪ Lack of organizational protocols ▪ Lack of protocols for evaluation of the performance of caregivers ▪ Lack of protocols for evaluation of the performance of organizations which offer in‐home care services
Ineffective interactions	▪ Ineffective and inefficient communication between in‐home caregivers ▪ Ineffective communication between in‐home caregivers and patients and their families
Inappropriate cultural/social context	▪ An ambivalent attitude to in‐home care ▪ Shortage of organizations which provide in‐home care ▪ Concentration of in‐home care organizations in big cities ▪ The government's failure to support patients who require in‐home care and home care organizations ▪ Lack of equipment and efficient personnel ▪ High costs of in‐home care
Professional issues	▪ Caregivers’ physical and psychological safety ▪ Occupational burnout ▪ Occupational stress

In this study, the forward‐backward method was used to translate quotations. The quotations were first translated into English by a bilingual person (fluent in Persian and English) and then translated again from English to Persian by another bilingual person. Researchers and some participants reviewed the text translated from English to Persian to make sure the meaning is the same.

### 3.1 Lack of effective and comprehensive standards

One of the extracted main themes was lack of effective and comprehensive standards which consists of the subthemes of lack of effective protocols for in‐home care and lack of organizational protocols (lack of protocols for evaluation of the performance of caregivers and lack of protocols for evaluation of the performance of organizations which offer in‐home care services).

#### 3.1.1 Lack of effective protocols for in‐home care

Based on the participants’ experiences, one of the barriers to efficient management of in‐home care is lack of effective protocols for giving care to patients at home. According to one of the participants:

“In in‐home care, what is missing is a clear established protocol which is based on scientific guidelines and evidence. Sometimes, I wonder if the procedures which I perform are scientific or not. There is a sense of indecision. I really don't know what instructions I should follow when I’m providing care” (P6).

#### 3.1.2 Lack of organizational protocols

Lack of organizational protocols, the other subtheme of lack of effective and comprehensive standards, consists of two subcategories: lack of protocols for evaluation of the performance of caregivers and lack of protocols for evaluation of the performance of organizations which offer in‐home care services.

#### 3.1.3 Lack of protocols for evaluation of the performance of caregivers

The participants’ experiences showed that another barrier to efficient management of in‐home care was lack of protocols for evaluation of the performance of caregivers. According to one of the participants:

“The performance of caregivers who are involved in in‐home care is not monitored on a precise and continuous basis. Anyone can do as she sees fit. Much of what my colleagues do is not based on nursing standards. There is no accurate supervision and even the organizations which provide in‐home care don't have a standard, scientific protocol for the evaluation of their personnel” (P9).

#### 3.1.4 Lack of protocols for evaluation of the performance of organizations which offer in‐home care services

Another issue in lack of effective standards was found to be lack of protocols to evaluate the performance of organizations which offer in‐home care services. As one of the participants stated:

“Many of the organizations which provide in‐home care do not have the required qualifications and I just don't know how they've managed to get the licence for giving in‐home care. On what basis are their activities evaluated? Some of the administrators at these institutes don't know the first thing about medical care and there is no supervision over their work” (P5).

### 3.2 Ineffective interactions

“Ineffective interactions” was another theme extracted from the data. It consists of ineffective and inefficient communication between in‐home caregivers and ineffective communication between in‐home caregivers and patients and their families.

#### 3.2.1 Ineffective and inefficient communication between in‐home caregivers

Based on the participants’ experiences, one of the barriers to efficient management of in‐home care is lack of effective inter‐professional communication between caregivers. According to one of the participants:

“On many occasions, I’ve seen conflict and disagreement between the personnel and the mangers of the institutes which provide in‐home care. The present work atmosphere is not friendly at all. The caregivers’ teamwork is poor and everyone wants to do things their own way. The head of the institute does not take any effective steps to resolve the conflicts and the situation is getting worse every day” (P4).

#### 3.2.2 Ineffective communication between in‐home caregivers and patients and their families

One of the most serious barriers to efficient management of in‐home care is lack of effective communication between in‐home caregivers on the one hand and patients and their families on the other. According to one of the participants:

“Effective communication is the heart of nursing and the key to solving problems and meeting patients’ needs. How can I expect my patients and their families to trust me when I can't establish a good and effective relationship with them?” (P8).

### 3.3 Inappropriate cultural/social context

Another barrier to efficient management of in‐home care was found to be lack of an appropriate cultural/social context which consists of the following subcategories: an ambivalent attitude to in‐home care, shortage of organizations which provide in‐home care, concentration of in‐home care organizations in big cities, the government's failure to support patients who require in‐home care and the organizations which provide in‐home care services, lack of equipment and efficient personnel, and the high costs of in‐home care.

#### 3.3.1 An ambivalent attitude to in‐home care

The participants’ experiences showed that there was an ambivalent attitude to in‐home care in the society: some patients and their families are open to receiving in‐home care services, while some other individuals have a negative outlook on these services. They do not trust home care organizations and do not want their patients to receive professional care at home. They mention that they can care for their patients at home and do not require the services provided by home are organizations. According to one of the participants:

“Some of the patients and their families don't feel good about home care. They think their dignity may not be preserved, their privacy may not be maintained, or their secrets may be given away” (P17).

#### 3.3.2 Shortage of organizations which provide in‐home care

Commenting on the number of home care organizations, one of the participants stated that:

“In our country, the role of home care institutes has not been established clearly yet and the number of the existing institutes is not nearly enough to meet the needs of patients and the society” (P13).

#### 3.3.3 Concentration of in‐home care organizations in big cities

According to one of the participants:

“Most of the available home care institutes and agencies are located in big cities and the people in small towns can't benefit from their services. It is necessary that the government and the policymakers in the healthcare system take steps to develop such centres in small towns too so the ideal of social justice in equitable access to medical care services can be realized” (P7).

#### 3.3.4 The government's failure to support patients who require in‐home care and home care organizations

The failure of senior healthcare administrators to support the patients who require in‐home care services and the organizations which provide those services was found to be another significant barrier to efficient management of in‐home care. According to one of the participants:

“The government is not giving the needed support to the patients who need in‐home care and the home care institutes. Many of these institutes go bankrupt and shut down because they can't afford the high cost of equipment and the salary of their personnel” (P10).

#### 3.3.5 Lack of equipment and efficient personnel

Based on the participants’ experiences, lack of equipment and experienced personnel is a major barrier to efficient management of in‐home care, with adverse effects on the quality of care. One of the participants stated that:

“At some home care centers, there is a lack of essential equipment. Some of their equipment is old and in need of repair. I’ve often had to use a suction machine to extract secretions from my patients’ airways, but the machine is old and doesn't work right. And when I tell our manager, he says, “New equipment will cost an arm and a leg and we can't afford it, so just use the one you have” (P2).

According to another participant:

“Home care organizations just don't care about the quality of care. To keep their expenses low, they hire inexperienced personnel. I’ve even seen them employ nursing students in some cases. In my opinion, this is blatant disregard for the patients’ rights and puts their safety at risk” (P18).

#### 3.3.6 High costs of in‐home care

The high costs of in‐home care pose another barrier to efficient management of in‐home care from the participants’ point of view. According to another participant:

“The cost of home care is really high. Unfortunately, insurance companies don't cover home care expenses and the patients themselves should pay the costs, which puts extra burden on the patients’ families. Very often, I’ve seen patients who needed several weeks of home care, but because they couldn't afford it, they decided to cancel their services after a week or two” (P11).

### 3.4 Professional issues

Another theme in significant barriers to efficient management of in‐home care was found to be “professional issues” which consists of the following subcategories: caregivers’ physical and psychological safety, occupational burnout and occupational stress.

#### 3.4.1 Caregivers’ physical and psychological safety

The participants’ experiences showed that another major barrier to efficient management of in‐home care was putting the caregivers’ physical and psychological safety at risk. The participants mentioned that since, unlike hospitals, there are not any security guards in the patients’ homes, patients and their families sometimes treat the caregivers disrespectfully or aggressively, which threatens the caregivers’ physical and psychological safety. According to one of the participants:

“I’ve seen several of my colleagues quit their jobs in home care because patients or their families insulted them and treated them with aggression” (P15).

#### 3.4.2 Occupational burnout

Based on the participants’ experiences, the nurses who provide in‐home care suffer from work overload. Caring for severely ill patients who require comprehensive care, for example patients with decreased consciousness and patients with spinal injuries, is a very demanding job and can lead to occupational burnout. According to one of the participants:

“Working in patients’ homes is very different and more difficult than working in the hospital. In the hospital, you can ask your colleagues to help you. But when it comes to home care, most of the time, one nurse has to do all the caring on her own and sometimes she has to work intensively in consecutive shifts. All this pressure causes fatigue and, in the long run, leads to occupational burnout. I myself stopped cooperating with home care companies because of the work overload” (P3).

#### 3.4.3 Occupational stress

The participants’ experiences showed that the nurses who provide in‐home care services are subject to considerable occupational stress and anxiety. As one of the participants stated:

“Caring for patients, especially those who are seriously ill, in their homes is so stressful. If the smallest thing happens to a patient, we really don't know who will defend our rights. Giving home care is taking a big risk and you should be prepared for the consequences” (P16).

## 4 DISCUSSION

The present qualitative study was conducted to identify the barriers to efficient management of in‐home care. The experiences of the in‐home care nurses who were interviewed in the present study show that the barriers to efficient management of in‐home care are comprised of lack of effective and comprehensive standards, ineffective interactions, inappropriate cultural/social context and professional issues. One of the major obstacles is lack of effective and comprehensive protocols, which consists of lack of effective protocols for in‐home care, lack of organizational protocols, lack of protocols for evaluation of the performance of caregivers and lack of protocols for evaluation of the performance of home care organizations.

In the study of Heydari et al. (2016), one of the major challenges in in‐home care is lack of executive protocols for caregivers who are involved in in‐home care. The participants of the study stated that there was no formal training for examining and prioritizing patients, nor were there any clear instructions about payment of salaries, estimation of cost or qualification of caregivers in the home care system. The participants believed that protocols could help resolve conflicts between caregivers and home care agencies and improve the quality of nursing services, which ultimately increases patient satisfaction (Heydari et al., 2016).

Similarly, in their study, Fatemi et al. (2019) report that there is a lack of clearly stated standards and list of duties of caregivers in in‐home care. Poor professional integrity, inadequate interaction between nurses and home care agencies, and lack of feedback from supervisors are reported to be other issues for in‐home care nurses. Lack of state guidelines is another challenge in nurses’ providing quality care (Fatemi et al., 2019). American Nurses Association (ANA) has laid down standards for home care nurses and their professional practice. Designed to assess home care, these standards address examination, diagnosis, identification of consequences, planning, implementation, evaluation, ethics, education, evidence‐based practice, quality of performance, communication, leadership, participation, assessment of professional performance, resource management and environmental hygiene. All home care centres are required to comply with these standards (Gorski, 2016).

In the United States and Australia, home care centres possess precise monitoring protocols and easy‐to‐use communication platforms. In many other countries, however, there is not a specific organization which is responsible for an accurate and thorough evaluation of the performance of home care companies (Palesy et al., 2018). In developed countries today, home care services are provided through smart, web‐based networks which entail various forms of comprehensive evaluation, monitoring and coordination in order to ensure information security, facilitate the registration of and access to patients’ medical history and reduce the cost of in‐home care (Valizadeh et al., 2019). Lack of a smart healthcare system in Iran is undeniable. Establishment of standard guidelines can improve the quality of home care services in Iran.

Another major barrier to efficient management of in‐home care is ineffective interactions which consist of ineffective and inefficient communication between in‐home care nurses and ineffective communication between in‐home caregivers and patients and their families. According to the study of Danielsen et al. (2018), lack of communication between doctors and patients following patients’ discharge puts patients’ safety at risk by preventing satisfactory home care planning. Thus, electronic communication between doctors and patients can increase patients’ safety after discharge (Danielsen et al., 2018). In the study of Beer et al. (2014), inadequate communication between home caregivers (nurses and doctors) is reported to result in nurses’ lack of access to information about patients’ medication regimen (Beer et al., 2014).

Sundler et al. (2016) state that one of the major challenges in communication between the in‐home caregivers of the elders is duty‐centred communication. Even though elderly patients have unique needs and concerns about their daily lives, their individual and existential issues are disregarded. Their caregivers do not try to establish emotional and non‐verbal communication with them or be active listeners, but simply administer the patients’ medication and hardly answer their questions (Sundler et al., 2016). The results of another study show that successful palliative home care depends on intimate communication and cooperation between patients, patients’ families, home care nurses and general practitioners (Arbaoui et al., 2012). It appears that in‐home caregivers can benefit from training in verbal and non‐verbal communication to understand their patients’ entire needs. Person‐centred communication and care‐related dialogs can improve the quality of in‐home care services (Li et al., 2016).

Another major barrier to efficient management of in‐home care was found to be lack of an appropriate cultural–social context for providing in‐home care. This theme consists of the subcategories of an ambivalent attitude to in‐home care, shortage of in‐home care organizations, concentration of in‐home care organizations in big cities, the government's failure to support patients who require in‐home care and the organizations which provide in‐home care services, lack of equipment and efficient personnel, and the high costs of in‐home care. According to Heydari et al. (2016), one of the major challenges in home care nursing is the society's distrust of non‐medical caregivers. In addition, the society's negative perception and distrust of doctors and healthcare providers’ lack of security are significant barriers to in‐home care. In‐home care nurses enter the personal territory of patients and their families, but do not have a pre‐determined care plan (Heydari et al., 2016). The results of the study of Heggestad et al. (2020) show that concern about failing to maintain patients’ privacy, disrespecting patients’ dignity and not showing respect for patients’ autonomy are important ethical challenges in in‐home care. Thus, in‐home care providers should observe the principles of professional ethics throughout their practice and respect their patients’ dignity while caring for them in order to encourage trust in home caregivers (Heggestad et al., 2020).

Financial difficulties pose another challenge to in home care nursing. The revenues of home care organizations often fall short of their running costs, and there is intense competition between these organizations. Social issues, lack of security and low social status are other problems which adversely affect nurses’ motivation to work in home care organizations (Danielsen et al., 2018). In‐home care nurses also refer to lack of human resources and equipment as obstacles in their profession (Association, 2013). According to a study, lack of insurance coverage restricts patients’ access to in‐home care services (Suurmond, 2016). In the one‐dimensional healthcare system of Iran, doctors’ monopoly on health care and the consequent disregard for non‐medical caregivers is another significant issue in home care. Moreover, another threat to in‐home care in Iran is the precedence of hospital services and doctors’ and nurses’ activities over secondary preventive services (Heydari et al., 2016). Lack of family support, language barriers, cultural differences and patients’ low levels of health awareness further complicate in‐home care. Additionally, home caregivers’ lack of specialized knowledge for providing care to all populations and the occasional need for several caregivers to care for a single patient can lead to interferences in roles and confuse patients (Guo et al., 2019).

Academic education, retraining courses and continuing education about in‐home care can help improve nurses’ performance in this area. Familiarizing patients and family caregivers with home care agencies and the duties of in‐home caregivers can facilitate the former's acceptance of and cooperation with in‐home caregivers. Healthcare organizations’ support of comprehensive insurance of patients can help reduce the financial burden of in‐home care. Also, allowing in‐home caregivers to participate in the management, administration and policymaking of healthcare organizations can help improve their social status.

The experiences of the participants in the present study showed that professional issues constitute another major obstacle to efficient management of in‐home care. Professional issues concern home caregivers’ physical and psychological safety, occupational burnout and occupational stress. Fatemi et al. (2019) mention physical and psychological stress as one of the main causes of caregivers’ reluctance to be involved in in‐home care.

The presence of patients’ family members at home, working alone and not receiving support from one's colleagues add to home caregivers’ stress. Work overload and physical and emotional exhaustion undermine the quality of home caregivers’ performance (Fatemi et al., 2019). According to the study of Yang (2017), home caregivers’ work environment is highly stressful as home caregivers are often subject to verbal aggression and other anxiety‐inducing behaviours on the part of their patients’ families, with potentially adverse effects on their performance (Yang, 2017). Yasin et al. (2020) report that home caregivers are more prone to occupational burnout than doctors and medical assistants because the former are in the frontline of caring for patients and, therefore, experience more emotional tension and burnout. Also, organizational stressors, including delayed salary payment, lead to home caregivers’ emotional exhaustion and occupational burnout (Yasin et al., 2020). Other stress‐inducing factors for in‐home care nurses are failure to be present at patients’ residences at a pre‐arranged date and time and fear of damaging patients’ property (Cooper et al., 2016). Other studies report poor work management, lack of peer support, shortage of equipment needed for providing in‐home care, low professional competence, unexpected events and inconvenient working hours to be causes of stress for home caregivers (Atashi & Nejatian, 2020). The study of Yoshimatsu and Nakatani (2020) shows that teamwork and cooperation between caregivers play a significant part in reducing home caregivers’ depression and stress (Yoshimatsu & Nakatani, 2020). In addition to supervision on the performance of home care organizations, support for the personnel and provision of the necessary equipment, the organizational culture should encourage all home caregivers to cooperate with each other. These conditions can increase in‐home caregivers’ psychical and psychological security and reduce the risk of stress, emotional exhaustion and occupational burnout for them.

## 5 IMPLICATIONS FOR NURSING

Nursing managers can use the results of this study to identify and eliminate the factors affecting the optimal implementation of home nursing care and improve the quality and effectiveness of home nursing services.

## 6 CONCLUSIONS

The in‐home care nurses who were interviewed in the present study had experienced a variety of obstacles in their career, which indicates the gap between the quality of in‐home care in Iran and the international home healthcare standards. Creating an appropriate cultural and social context in Iranian societies, providing a suitable infrastructure, including insurance coverage, clear, comprehensive guidelines for in‐home care, promoting teamwork and organizing workshops to encourage effective communication between caregivers and patients can help improve the quality of in‐home care in Iran.

## CONFLICTS OF INTEREST

The authors declare that they have no competing interests.

## AUTHOR CONTRIBUTIONS

Mostafa Bijani, Fateme Mohammadi, and Banafsheh Tehranineshat, Study design and Manuscript preparation. Mostafa Bijani, Fateme Mohammadi, and Parisa Sabetsarvestani Data collection. Fateme Mohammadi, Zhila Fereidouni, Banafsheh Tehranineshat, Mostafa Bijani, Data analysis. All authors read and approved the final manuscript.

## Data Availability

The data that support the findings of this study are available from the corresponding author upon reasonable request.
